# Special Issue “Research in iPSC-Based Disease Models”

**DOI:** 10.3390/ijms27104489

**Published:** 2026-05-17

**Authors:** Eric Deneault

**Affiliations:** Regulatory Research Division, Centre for Oncology, Biostatistics, Research and Radiopharmaceuticals, Biologic and Radiopharmaceutical Drugs Directorate, Health Products and Food Branch, Health Canada, Ottawa, ON K1A 0K9, Canada; eric.deneault@hc-sc.gc.ca

## 1. Introduction

Biomedical research has been transformed by the discovery of human induced pluripotent stem cells (iPSCs), providing unlimited access to patient-specific human cell types for disease modeling and mechanistic studies. In the last 20 years, significant improvements have been made in differentiation protocols to generate multicellular, three-dimensional (3D), and genetically engineered systems capable of recapitulating complex human disease features.

This Special Issue, “Research in iPSC-Based Disease Models”, showcases six publications that illustrate the expanding scope of iPSC technologies across various organs and diseases (Table 1).

These contributions encompass neurological, cardiovascular, musculoskeletal, and craniofacial disorders. They also highlight promising methodological innovations such as organoids, inducible reporter systems, proteomics, and 3D tissue constructs. Collectively, these studies reveal how iPSC-derived models are progressing into powerful tools for dissecting disease mechanisms and facilitating translational applications. This Editorial summarizes the key findings of the Special Issue and discusses their broader implications in the field of iPSC-based disease modeling.

## 2. Neuroinflammation and Neurodegeneration in iPSC-Derived Neural Systems

**Figure 1:**
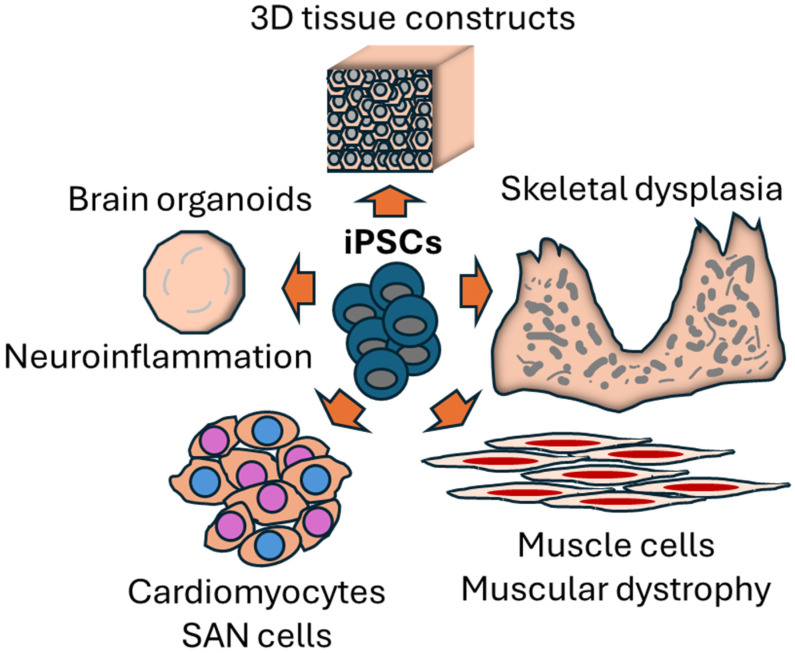
Various types of differentiated cells are derived from iPSCs in this Special Issue.

It has been postulated that neuroinflammation represents a potential driver of neurological disorders [1]. However, the study of inflammatory signaling in living human brains remains challenging and almost impossible. El Sabbagh et al. addressed this problem by establishing a new method to investigate the role of NLRP3 inflammasome activation in cerebral organoids (Contribution 1, Table 1). In this study, cerebral organoids, derived from iPSCs (Figure 1), recapitulated different aspects of 3D brain architecture and cellular diversity. These organoids provided a more physiologically relevant context than conventional 2D cultures.

The authors managed to adapt models of inflammasome activation, previously developed in peripheral immune cells, to cerebral organoids (Contribution 1, Table 1). This advance facilitates the visualization of apoptosis-associated speck-like protein containing a CARD (ASC) speck formation and downstream inflammatory signaling within neural cells. Since NLRP3 activation has previously been associated with mitochondrial dysfunction and neurological disorders such as Alzheimer’s disease [2], Parkinson’s disease [3], and bipolar disorder [4], this approach provides a valuable method to investigate neuroinflammatory mechanisms in patient-derived brain models. In perspective, these results illustrate how 3D organoid techniques could bridge the gap between cellular assays and in vivo neurobiology.

Complementing this approach, Gandy et al. developed an inducible bioluminescent tagging system to study Parkinson’s disease (PD)-associated genes in iPSCs and downstream neural cells (Contribution 2, Table 1). In this study, the clustered regularly interspaced short palindromic repeat (CRISPR) gene editing system was used to append the luminescent subunit HiBiT to endogenous PD genes, and the expression of the corresponding subunit LgBiT was induced upon doxycycline treatment. This sensitive live-cell assay enabled real-time quantification and localization of endogenous PD proteins, without the use of any antibody. Using this approach, various PD-related genes were successfully tagged, including *GBA1*, *GPNMB*, *LRRK2*, *PRKN*, *SNCA*, *VPS13C* and *VPS35* (Contribution 2, Table 1). This work addresses a major bottleneck in neurological research, i.e., the lack of high-quality antibodies and tools to detect low-expression proteins. It also highlights the potential of endogenous tagging methods for functional genomics in iPSC-derived neurons.

Together, these two studies shed light on complementary approaches in neurological disease modeling, i.e., increasing tissue culture complexity through organoids and increasing molecular precision through genome engineering.

## 3. Cardiovascular Differentiation and Sex-Dependent Biology

Cardiovascular diseases represent the main cause of mortality in humans, but several corresponding pathologies are currently poorly modeled in the literature [5]. From this perspective, Sleiman et al. reviewed recent innovations in differentiating sinoatrial node (SAN)-like cardiomyocytes from human iPSCs (Figure 1), representing an important step in modeling cardiac pacemaker disorders (Contribution 3, Table 1). SAN-specific differentiation represents a technical challenge since most iPSC-derived cardiomyocytes look like ventricular or atrial subtypes [6]. This review assesses different signaling pathways leading to pacemaker differentiation, including modulation of WNT, BMP, and NODAL pathways (Contribution 3, Table 1). The authors suggest that SAN-like cardiomyocytes might allow disease modeling of arrhythmias that are not captured by standard cardiomyocyte populations. Moreover, the idea of cell-based biological pacemakers supports the translational potential of iPSC-derived cardiac cell types for regenerative medicine.

A complementary model on cardiovascular disease is presented by Ariyasinghe et al., who explored sex-based proteomic differences in iPSC-derived vascular smooth muscle cells (iVSMCs) (Contribution 4, Table 1). This article addresses the mechanisms of sex differences in the occurrence of cardiovascular disease that are often observed in the clinic. Using quantitative proteomics, the authors showed that iVSMCs are similar to primary vascular smooth muscle cells and present sex-dependent variations in adhesion and metabolic pathways that are observed in vascular disease. In addition to underlining the biological relevance of differentiated cells, this study highlights the importance of specific genetic background variables such as sex. This work identified signaling pathways underlying sex-specific vascular phenotypes, thus supporting the use of iPSC models for therapeutic development.

## 4. Musculoskeletal and Craniofacial Disorder Modeling Using iPSC Differentiation and 3D Structures

In addition to neural and cardiovascular tissues, iPSCs can generate musculoskeletal and craniofacial cells. In this Special Issue, Lisowska et al. establish an iPSC-derived muscle model of Emery–Dreifuss muscular dystrophy type 1 (EDMD1), a genetic disorder caused by mutations in *EMD*, coding for the nuclear envelope protein emerin (Contribution 5, Table 1). These authors differentiated patient-specific mutant iPSCs during multiple myogenesis steps and generated myogenic progenitors, myoblasts, and multinucleated myotubes that recapitulated muscle-relevant phenotypes (Figure 1). This powerful model overcomes important limitations in EDMD1 research, such as limited patient tissue availability and species differences in animal models. It also provides a helpful platform to study muscle disease mechanisms across diverse developmental stages. More specifically, this system has shown disrupted differentiation and structural abnormalities in EMD-deficient muscle cells. These results illustrate well how patient-specific iPSCs can reveal pathogenic features that are not easily detectable in animal models.

In a similar but distinct lineage context, Kondo et al. developed a new iPSC-derived 3D hard tissue construct to model hereditary tooth and skeletal dysplasia (Contribution 6, Table 1). Leveraging mesoderm differentiation with osteogenic and odontogenic signals under 3D culture conditions, the authors created mineralized 3D constructs expressing osteogenesis and dentinogenesis markers. Since these structures were derived from hypophosphatasia patient-specific iPSCs (Figure 1), they reproduced key disease phenotypes, such as those with reduced mineralization and differentiated marker expression. This study highlights the importance of 3D culture systems in modeling developmental disorders that imply intricate tissue architecture. Moreover, the capacity to monitor both osteogenesis and dentinogenesis simultaneously in patient-specific 3D constructs represents a significant innovation for musculoskeletal and craniofacial disorder modeling.

## 5. Multidisciplinary Technological Advances in Disease Modeling Using iPSCs

The works presented above encompass the study of diverse tissues and diseases. A few important themes have emerged from them. For example, the integration of precise gene engineering may facilitate specific molecular readouts. The inducible HiBiT-LgBiT tagging system illustrates how CRISPR-enabled endogenous reporters can empower quantitative live-cell analysis of PD-associated proteins (Contribution 2, Table 1). Similar gene editing approaches are set to expand to other disease models. The dynamic monitoring of various cellular signaling pathways can accelerate the discovery of more efficient therapeutics.

Moreover, increasing structural complexity was commonly observed in research models in these studies. The cerebral organoids grown by El Sabbagh et al. (Contribution 1, Table 1), as well as the 3D mineralized constructs presented by Kondo et al. (Contribution 6, Table 1), exemplify progression from monolayer cellular cultures toward organ-like systems that better recapitulate complex in vivo configuration and cell–cell interactions. These models support the study of the multicellular interactions that are at play during inflammation and extracellular matrix deposition or even during differentiation gradients.

Lineage-specific differentiation of iPSCs is also shared between different studies presented above. It involves the development of specific procedures in different models. For instance, the generation of SAN-like cardiomyocytes (Contribution 3, Table 1) or stage-specific myogenic cells (Contribution 5, Table 1) demonstrates the importance of guided differentiation towards distinct disease-relevant mature cells. Unique cell identity is critical for modeling different disorders that affect highly specialized tissues.

Furthermore, the identification of gender-specific proteomic differences in iVSMCs (Contribution 4, Table 1) illustrates the importance of considering genetic background differences in iPSC research. As the number of iPSC biobanks increases and gene editing therapies expand, precision medicine will need to be supported by an improved integration of gender, ancestry, and patient-specific genotypes.

## 6. Translational Implications of iPSC-Based Models

The studies presented in this Special Issue highlight the translational possibilities of iPSC-based disease models. For example, patient-specific cerebral organoids (Contribution 1, Table 1) and muscle cells (Contribution 5, Table 1) can offer interesting opportunities for mechanistic studies and large drug screenings. Likewise, SAN-like cardiomyocytes (Contribution 3, Table 1) and mineralized 3D hard tissue constructs (Contribution 6, Table 1) underline the potential of cell-replacement and regenerative therapies. Furthermore, inducible reporter systems (Contribution 2, Table 1) may facilitate drug target validation and pharmacodynamic monitoring.

In spite of these considerable advances, scientists still face numerous challenges. Indeed, cultured cells directly differentiated from iPSCs may present various states of maturation and function. This variability tends to limit the application of some cell therapies, particularly for adult-onset diseases such as PD. Improved standardization of differentiation protocols and reproducibility between distinct laboratories will offer solutions to address these concerns. Moreover, organoid systems need to integrate a wider range of cell types, such as immune, vascular, and stromal cells, in order to achieve physiological tissue complexity.

The integration of CRISPR gene editing technologies into these iPSC models will continue to improve the translational potential by offering the creation of isogenic controls, where genetic background variability is reduced and causal effect is strengthened. CRISPR can provide access to multiplex engineering and allow the introduction of specific mutations, corrections, reporters for high-content screens, and diverse co-occurring edits to model complex genetic diseases. It is also compatible with single-cell and multi-omics analyses to facilitate the assessment of heterogeneity and genotype–phenotype relationships.

In the future, a combination of several technological advances presented in this Special Issue will likely lead progress in iPSC-based models. For example, gene-edited reporter systems could be integrated into 3D organoids to study various disease pathways. Large-scale multi-omics analyses including proteomics could be applied to the lineage-specific differentiation of iPSCs to accelerate therapeutic target discoveries.

Collectively, the six contributions to this Special Issue (Table 1) illustrate the broad application of iPSC-based disease modeling across neurological, cardiovascular, muscular, and craniofacial systems. Such innovations in organoid models, reporter engineering, gene editing, lineage-specific differentiation, proteomics, and 3D hard tissue constructs converge to generate more physiologically relevant human disease models. These tools increase our understanding of disease mechanisms but also speed up the development of regenerative cell therapies. The impact of iPSC technologies will continue to evolve, especially in the presence of emerging fields such as artificial intelligence-based analysis of complex data. The translational potential of current iPSC research underscores its pivotal role in the future of biomedical science.

## Figures and Tables

**Table 1:** Contributions to this Special Issue.

Title	Publication Type	Authors	Contribution ID
A Dynamic Protocol to Explore NLRP3 Inflammasome Activation in Cerebral Organoids	Protocol	Dana El Soufi El Sabbagh, Liliana Attisano, Ana Cristina Andreazza, Alencar Kolinski Machado	1
An Inducible Luminescent System to Explore Parkinson’s Disease-Associated Genes	Article	Anelya Gandy, Gilles Maussion, Sara Al-Habyan, Michael Nicouleau, Zhipeng You, Carol X.-Q. Chen, Narges Abdian, Nathalia Aprahamian, Andrea I. Krahn, Louise Larocque, Thomas M. Durcan and Eric Deneault	2
Differentiation of Sinoatrial-like Cardiomyocytes as a Biological Pacemaker Model	Review	Yvonne Sleiman, Jean-Baptiste Reisqs and Mohamed Boutjdir	3
Identification of Disease-Relevant, Sex-Based Proteomic Differences in iPSC-Derived Vascular Smooth Muscle Cells	Article	Nethika R. Ariyasinghe, Divya Gupta, Sean Escopete, Deepika Rai, Aleksandr Stotland, Niveda Sundararaman, Benjamin Ngu, Kruttika Dabke, Liam McCarthy, Roberta S. Santos, Megan L. McCain, Dhruv Sareen and Sarah J. Parker	4
Human iPSC-Derived Muscle Cells as a New Model for Investigation of EDMD1 Pathogenesis	Article	Marta Lisowska, Marta Rowinska, Aleksandra Suszynska, Claudia Bearzi, Izabela Laczmanska, Julia Hanusek, Amanda Kunik, Volha Dzianisava, Ryszard Rzepecki, Magdalena Machowska and Katarzyna Piekarowicz	5
Fabrication of Hard Tissue Constructs from Induced Pluripotent Stem Cells for Exploring Mechanisms of Hereditary Tooth/Skeletal Dysplasia	Article	Takeru Kondo, Sermporn Thaweesapphithak, Sara Ambo, Koki Otake, Yumi Ohori-Morita, Satomi Mori, Naruephorn Vinaikosol, Thantrira Porntaveetus and Hiroshi Egusa	6
